# A Cognitive Model of Pathological Worry in Children and Adolescents: A Systematic Review

**DOI:** 10.1007/s10567-020-00311-7

**Published:** 2020-01-28

**Authors:** Annabel Songco, Jennifer L. Hudson, Elaine Fox

**Affiliations:** 1 Department of Experimental Psychology, University of Oxford, Oxford, UK; 2 Centre for Emotional Health, Department of Psychology, Macquarie University, Sydney, Australia

**Keywords:** Worry, GAD, Children, Adolescents, Cognitive model

## Abstract

Worry is common in children and adolescents, yet some youth experience excessive worries that persist over time and cause significant distress. Whilst the literature on worry and generalised anxiety disorder (GAD) in adults is well established, relatively less is known about the cognitive mechanisms underlying child and adolescent worry. An influential cognitive model of adult pathological worry (Hirsch and Matthews in Behav Res Therapy 50:636–646, 10.1016/j.brat.2012.06.007, 2012) proposes that negative information-processing biases, reduced executive functions, and verbal worry are critical in the aetiology of GAD in adults. The current systematic review investigated whether this cognitive model of worry could be extended to understand child and adolescent worry. Following a systematic search of the literature and screening for eligibility, 30 studies were identified. Evidence indicates that negative information-processing biases and reduced executive functions play an important role in worry and GAD in children and adolescents. However, evidence that children and adolescents experience verbal worry is inconclusive. Building upon Hirsch and Matthews' cognitive model (Behav Res Therapy 50:636–646, 10.1016/j.brat.2012.06.007, 2012), we propose a model of child and adolescent worry to provide a guiding framework for future research. We conclude that cognitive models of worry should incorporate a developmental framework in order to provide greater insight into the mechanisms uniquely associated with worry in children and adolescents and help to identify the cognitive processes to target for early interventions and treatments.

## Child and Adolescent Worry

Worry is a cognitive component of anxiety (Borkovec et al. 1991) that involves repetitive thoughts and images that focus on the potentially negative outcomes of future events (Vasey and Daleiden, 1994). Research shows that worry is common in children and adolescents and varies continuously across the normal population (Goncalves and Byrne 2013). In moderation, worry can serve as an adaptive process that enables problem-solving, prepares individuals for future threat, and increases motivation (Davey 1994). However, at the other end of the spectrum, pathological worry is of clinical concern and is characterised by excessive worries that persist over time and cause significant distress.

Pathological worry in children and adolescents is associated with poor academic functioning, school absenteeism, severe difficulty concentrating, withdrawal from social activities, and disrupted sleep patterns (Albano and Hack 2004). The content of worries in children and adolescents is wide ranging and typically involves issues relating to school, relationships, health, as well as interpersonal and social problems (Muris et al. 1998; Silverman et al. 1995). Females tend to report higher levels and frequencies of worry compared with males (Barahmand 2008; Caes et al. 2016; Muris et al. 2001). In one study, for instance, a gender difference in worry frequency had emerged by age ten, but was not present at age seven, whilst gender differences in the interference caused by worry had emerged by age 13. Pubertal development in females was associated with worry frequency, with those 13-year-olds with advanced pubertal timing experiencing greater worry frequency (Caes et al. 2016).

Excessive levels of worry, when left untreated, is a risk factor for the development of anxiety disorders, with many adults reporting that their excessive worries began to develop in childhood or adolescence (Costello et al. 2005; Pine et al. 1998). Pathological worry is one of the core features of generalised anxiety disorder (GAD) (American Psychiatric Association 2013) and lifetime prevalence of GAD amongst youth ranges from 2 to 6%, with early onset occurring from middle childhood onwards (Merikangas et al. 2010). Thus, pathological worry is a debilitating mental health problem in children and adolescents with long-term negative consequences.

Childhood and adolescence, collectively referred to as youth from here onwards, is a period that entails significant cognitive, social, and physiological changes that can have an impact on the development of worry (Copeland et al. 2014). Recent studies in adolescents show heightened sensitivity and neuroplasticity in brain development, where certain cognitive skills, thought patterns, and behaviours are particularly malleable (Fuhrmann et al. 2015). Therefore, adolescence may represent a vulnerable period, following childhood, which offers a unique opportunity to turn the development and maintenance of worry around in its early stages. Despite the early onset and the negative consequences associated with high worry in youth, research examining the cognitive mechanisms underlying the aetiology of worry in children and adolescence remains scarce.

## Developmental Models of Worry

Few developmental models highlight the cognitive pathways that lead to pathological worry in children and adolescents (Kertz and Woodruff- Borden 2011; Vasey 1993). However, cognitive processes are likely to be important. For example, Vasey’s influential model of worry (1993) proposes that the capacity, complexity, and elaboration of worry increases from middle childhood, at the age of eight onwards, as children develop cognitive skills such as deductive reasoning that enable them to anticipate future events and elaborate on threatening possibilities. Muris et al. (2002) found that although children as young as three reported worrying, children who successfully passed Piaget’s conservation task (an indication of a child’s ability to consider different aspects of an event or situation) were more likely to have increased worries. Although worry requires the child to be able to predict at least one negative outcome from a future event, the more a child is able to generate possible negative outcomes, the greater the child’s likely capacity to worry. In addition, Vasey proposes that the ability to switch from mental imagery to verbal worry is a cognitive skill that develops with age and this transition occurs around middle childhood. During adolescence, worry becomes more prominent with the development of abstract thinking and, as mentioned, the cognitive ability to foresee multiple negative outcomes (Vasey and Daleiden 1994). Therefore, whilst some evidence does support the role of cognitive maturation in children’s experience of worry (Muris et al. 2000, 2002), further research is needed to uncover the cognitive factors associated with the development of worry throughout childhood and adolescence.

The complex interplay between genetics, temperament, cognitions, emotion, and parental risk factors in the development of pathological worry is captured in a comprehensive model proposed by Kertz and Woodruff-Borden (2011). They highlight a range of vulnerability factors and potential pathways that may contribute to the aetiology of worry in youth. These include the importance of cognitive risk factors such as information-processing biases, intolerance of uncertainty, and metacognitive beliefs about worry. Whilst there is substantial evidence supporting the role of genes, child temperament, parental factors, and environmental influences in the transmission of pathological worry (Aktar et al. 2017; Hudson and Rapee 2004), it is only recently that research has begun to examine the cognitive pathways associated with worry or GAD in youth. Therefore, the scope of this systematic review is to investigate cognitive risk factors, particularly information-processing biases and executive functions associated with worry in children and adolescents, which remains a largely understudied area.

## Cognitive Factors Associated with Worry

The critical role of cognitive factors in the aetiology of worry is well established in adults. For example, the avoidance of processing threats in the form of imagery due to their greater emotional impact (Borkovec 1994), maladaptive beliefs about the benefits and detriments of worry (Wells 1995), intolerance of uncertainty (Dugas et al. 1998), negative information-processing biases in combination with deficits in attentional control (Eysenck et al. 2007; Hirsch and Mathews 2012), lack of problem-solving confidence (Davey 1994), deficits in regulating emotions (Mennin et al. 2005), and high levels of emotional reactivity (Newman and Llera 2011) have all been shown to be associated with heightened worry. Several theoretical models have emerged that are based on these findings. For instance, the Avoidance Model of Worry and GAD (Borkovec 1994), the Metacognitive Model of worry (Wells 1995), the Intolerance of Uncertainty Model (Dugas et al. 1998), the Cognitive Model of Pathological Worry (Hirsch and Matthews 2012), and the Emotion Dysregulation Model (Mennin et al. 2005), have all contributed to a deeper understanding of worry in adults and facilitated the development of new treatments for GAD (see Behar et al. 2009 for review). Whilst other biological and environmental risk factors contribute to worry, overall cognitive models remain the dominant approach for explaining the underlying processes that cause and maintain worry and GAD.

In contrast, less is known about the defining characteristics of worry and GAD in children and adolescents. The absence of a developmentally appropriate understanding of worry often results in adult conceptualisations of worry being applied to youth (Cartwright-Hatton et al. 2011). There is some evidence to suggest that adult models of GAD may also be applicable to child and adolescent worry. For instance, intolerance of uncertainty, a central feature of the Intolerance of Uncertainty model of GAD (Dugas et al. 1998), is an important risk factor associated with worry in youth. Studies have shown that youth with high worry and GAD typically demonstrate greater levels of intolerance of uncertainty, relative to non-anxious youth (see Osmanağaoğlu et al. 2018 for review). In addition, the role of metacognitive beliefs, as outlined in Well’s Metacognitive Model of GAD (1995), has been extensively examined in child and adolescent worry (see Ellis and Hudson 2010 for review). Whilst negative metacognitive beliefs about the harmful nature of worry are associated with high worry in youth, contrary to the adult literature, there have been mixed findings that endorsing positive beliefs about the usefulness of worry are associated with high worry. Broadly, these findings suggest that cognitive processes similar to those in adults may also influence child and adolescent worry. However, it is not clear to what extent adult cognitive models of GAD are fully applicable to youth, given the developmental changes experienced throughout childhood and adolescence.

Whilst some cognitive processes such as intolerance of uncertainty and metacognitive beliefs have been shown to contribute to child and adolescent worry, the role of information-processing biases and executive functions in child and adolescent worry remains a relatively neglected area of empirical enquiry. However, based on theoretical perspectives and evidence from the adult literature (Hirsch and Matthews 2012), it seems reasonable to hypothesise that information-processing biases may be an important cognitive pathway to pathological worry and GAD in adolescents. Therefore, the aim of this systematic review is to investigate what is known about the role of information-processing biases and executive functions in child and adolescent worry, drawing from the theoretical framework of Hirsch and Matthews’ cognitive model of pathological worry (2012). Whilst we acknowledge that a wide range of other cognitive factors are likely to contribute to child and adolescent worry, we argue that information-processing biases and executive functions are likely to be central to the worry process in youth and therefore further investigation is warranted. Before reviewing the youth literature, we will examine Hirsch and Mathews’ (2012) model of adult worry in more detail.

## Hirsch and Matthews’ Cognitive Model of Pathological Worry

Hirsch and Matthews’ cognitive model of pathological worry (2012) provides a strong evidence-based framework highlighting the critical role that information-processing biases, executive functions, and the verbal processing of worry all play in the development and maintenance of worry in adults. Particular combinations of these three building blocks result in a potent form of pathological worry, as seen in GAD, that is generalised, excessive, and uncontrollable. In addition to these three main component processes, the Hirsch and Matthews’ model also proposes that pathological worry is maintained by means of other cognitive factors such as intolerance of uncertainty, emotion dysregulation, and maladaptive beliefs. The applicability of Hirsch and Matthews’ cognitive model to understanding child and adolescent worry is yet to be examined and, we argue, could provide valuable insights into the cognitive mechanisms underlying worry in youth.

There is strong empirical evidence supporting key aspects of Hirsch and Matthews’ cognitive model (2012) with regard to adult worry. First, negative cognitive biases have been shown to play an important role in the aetiology of adult worry and GAD (Mathews and MacLeod 2005) and are mechanisms that cause and maintain psychopathology (Beck et al. 1985, 1979; Williams et al. 1997). Adults with high levels of worry or GAD demonstrate selective attention towards threat (Hayes et al. 2010; MacLeod et al. 1986), are slower to disengage away from threat (Fox et al. 2001), and have a tendency to interpret ambiguous scenarios as threatening (Hirsch et al. 2009). Hirsch and Matthews propose that these negative cognitive biases are relatively involuntary ‘bottom-up’ processes responsible for negative thoughts that eventually intrude into awareness.

The second building block outlined in Hirsch and Mathews’ cognitive model is deficits in the central executive function of working memory or attentional control. In contrast to cognitive biases, executive functions involve voluntary ‘top-down’ processes associated with prefrontal cortical structures. Experimental studies in adults have shown that worry and GAD are associated with reduced attentional control and working memory capacity (Eysenck et al. 2007; Fox et al. 2015; Hayes et al. 2008; Leigh and Hirsch 2011; Moran 2016). The assumption is that worry is responsible for drawing attentional control resources and working memory capacity away from other tasks, thus impairing the ability to redirect worrisome thoughts.

Hirsch and Matthews propose that worry arises from an interaction between ‘bottom-up’ involuntary processes, such as attention and interpretation biases towards threatening information, and ‘top-down’ voluntary processes in the executive control of attention. In individuals not prone to worry, when a threatening thought intrudes into awareness, effortful control processes operate efficiently to inhibit the further representation of threat. In marked contrast, for individuals prone to high worry, negative intrusions are more likely to enter into awareness due to the stronger influence of pre-existing negative cognitive biases and habitual thought patterns alongside a depleted control of attention. Consequently, a worry episode develops in the form of verbal thoughts that ultimately become excessive and uncontrollable.

The predominantly verbal nature of worry represents the third component process of Hirsch and Matthews’ cognitive model. The model proposes that pathological worry is characterised by verbal thoughts that are relatively non-specific and general. The authors suggest that this verbal-linguistic form of worry is more potent than imagery-based worry as it gives rise to abstract negative outcomes that are typically vague and difficult to resolve. Studies in adults have found that verbal worry functions as a form of cognitive avoidance of the negative outcomes evoked by mental imagery (Borkovec and Inz 1990) and increases subsequent negative intrusions compared to imagery-based worry (Butler et al. 1995; Leigh and Hirsch 2011; Stokes and Hirsch 2010).

Whilst a large body of empirical work supports Hirsch and Matthews’ cognitive model of pathological model in adults, relatively few studies have investigated how these cognitive processes might operate during worry in children and adolescents. First, the relationship between cognitive biases and child and adolescent worry remains largely unexplored, with the majority of research focused on anxiety and mood disorders (Crick and Dodge 1994; Muris and Field 2008; Lau and Waters 2016; Platt et al. 2016). Second, whilst a growing body of evidence indicates that impairments in attentional control and working memory are associated with greater levels of anxiety in youth (Kertz et al. 2016; Vilgis et al. 2015), few studies have examined this directly in relation to worry. Finally, it is unclear whether the nature of worry in youth reflects the same verbal processes as those observed in adults, with Vasey’s developmental model (1993) proposing that this transition occurs during middle childhood.

This is a clear gap in the literature and to date, no systematic review has investigated the role of cognitive biases and executive functions in the transdiagnostic factor of worry in children and adolescents. Therefore, the objective of the current review is to examine existing evidence in children and adolescents for the three building blocks outlined in Hirsch and Mathew’s (2012) cognitive model of adult pathological worry and evaluate the applicability of this model to understanding worry in youth. In line with the first building block, the current review investigated evidence for the association between worry and attention, interpretation, and memory biases in children and adolescents. These cognitive biases, featured in information-processing models of anxiety in youth, are hypothesised to play a key role in the aetiology of childhood anxiety (Crick and Dodge 1994; Muris and Field 2008). In addition, the review examined evidence in youth of an association between worry and attentional control, working memory, and the verbal processing of worry as outlined in the second and third building blocks of Hirsch and Matthews’ cognitive model.

Whilst we acknowledge that there are a wide range of cognitive factors that contribute to child and adolescent worry, we focus specifically on Hirsch and Matthews’ cognitive theory of pathological worry in order to investigate the association of cognitive biases, executive functions, and verbal worry in youth with high worry or GAD, as these have been shown to be vital to adult worry. To our knowledge, this is the first systematic review to evaluate the applicability of Hirsch and Matthews’ cognitive model of worry to younger populations. A deeper understanding of how these cognitive mechanisms operate in child and adolescent worry would provide a greater insight into the psychological processes to target in treatments and early interventions. Following our systematic review of the literature on the cognitive building blocks of youth worry, we outline an extended cognitive model of pathological worry that is applicable to child and adolescent populations to help guide future research.

## Method

### Procedure

A systematic review of the literature was conducted in April 2019 following the PRISMA guidelines (Moher et al. 2009). Studies were identified by searching electronic databases, scanning references and citations of articles, and consultation with experts in the field. The search was applied to the PsycINFO (1987—April 12, 2019), MEDLINE (1974—April 12, 2019), EMBASE (1946—April 12, 2019), and PubMed electronic databases. A range of subject headings and search terms were used to obtain articles relevant to the three building blocks in the cognitive model of pathological worry (Hirsch and Matthews 2012) in relation to children and adolescents. Therefore, the search comprised of key terms was related to the following: *Information-processing biases* (“cog* bias*” or “cog* process*” or “cog* process* bias*” or “cog* factor*” or “information process* bias*” or “emotion process* bias*” or “cog* model*” or “information process* model*” or “attention* bias*” or “interpret* bias*” or “memory bias*”); *Executive functions* (“attention* control” or “executive function*” or “cog* control” or “inhibit* control” or “executive control” or “emotion* control” or “working memory” or “inhibition” or “updating”); and *Verbal processing of worry* (“verbal” or “verbal process*” or “intrusive thought*). These search terms were all paired with the terms *Worry* (“worry” or “generalised anxiety disorder*” or “GAD”) and *Youth* (“child*” or “adolescen*” or “youth”).

**Fig. 1:**
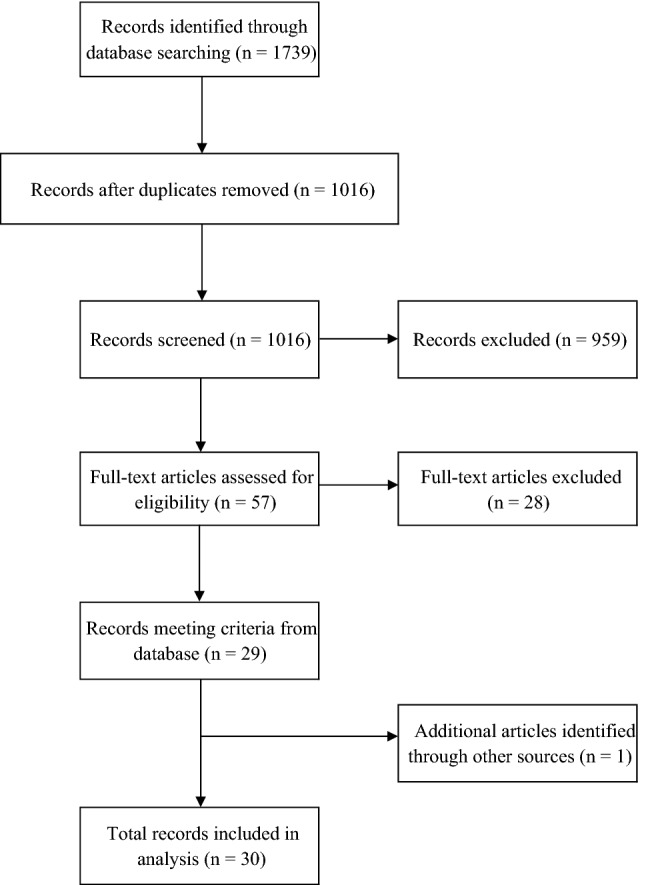
PRISMA diagram of selection of studies

In the initial search, 1739 articles were retrieved. After duplicates were removed, the titles and abstracts of 1016 articles were screened for relevance and 57 full-text articles were retrieved for further screening. The full-text articles were screened and resulted in 29 studies from the database that were eligible for inclusion. One additional article was identified by searching through relevant journals, which resulted in 30 studies included in the systematic review (see Fig. 1). In addition, the authors performed a quality assessment of each study using an established quality assessment tool (Effective Public Health Practice Project 1998). This was conducted to assess the risk of bias and discrepancies between the authors. Any discrepancies were resolved through discussion.

Studies were eligible for inclusion in the current systematic review if they examined information-processing biases, executive functions, and the verbal nature of intrusive thoughts in relation to worry or GAD in children and adolescents. The inclusion criteria included children and adolescents up to the age of 18, clinical and non-clinical studies that used a standardised measure of worry or GAD, clinical studies that included participants with a diagnosis of GAD, and studies that reported outcomes specific to GAD in comparison with non-anxious or non-GAD groups. This decision was made in order to address the specificity of worry relevant to Hirsch and Matthews’ cognitive model, that is, pathological worry in GAD, as opposed to overall anxiety. The upper age limit of 18 years was selected to provide a broad age range to include the typical age of adolescents in their final years of secondary school education. Further criteria included articles published in peer-reviewed journals and studies available in English. Review papers and opinion pieces were excluded from the systematic review. The papers identified in the systematic search comprised a variety of research questions and a wide range of methods and measures were used. Therefore, a qualitative synthesis or meta-analysis of the data was not feasible or appropriate. Instead, a narrative synthesis of the findings is presented below.

## Results

Thirty studies in the child and adolescent literature investigated at least one of the three key components of Hirsch and Matthews’ cognitive model. Tables 1, 2, and 3 provide a summary of the studies reviewed. Overall, the quality assessment of articles in the systematic review was moderate to strong, indicating a low risk of bias (see Table 4 in "Appendix").

### Information-processing Biases

#### Attention Bias

Eight studies examined the association between attention bias and worry or GAD in youth (see Table 1). The studies showed that children and adolescents (7–18 years old) with GAD displayed an attention bias towards threat-related words, relative to neutral words, in a Dot-probe task (Dalgleish et al. 2003; Taghavi et al. 1999). Similarly, Dot-probe studies using visual stimuli in younger samples of 7–12-year-olds (Waters et al. 2008) and 5–13-year-olds (Waters et al. 2014) found that youth with GAD have an attention bias towards threat-related angry faces, relative to neutral faces. In all studies, this pattern of selective attention towards threatening stimuli was specific to youth with GAD in comparison with youth with other disorders and healthy controls, which suggests that negative attention biases play a role in pathological worry in youth.

However, some studies using the visual Dot-probe task have shown mixed results. One study with 7–18-year-olds found that attention bias towards threat was associated with overall anxiety symptoms, although no relationship between threat bias and a diagnosis of GAD, separation anxiety, or social phobia emerged (Roy et al. 2008). Similarly, another study showed that threat bias was positively associated with social anxiety and social phobia, but not with GAD, panic or separation anxiety in 6–18-year-olds (Abend et al. 2017). Furthermore, the results were not moderated by age or gender. Both studies utilised samples drawn from multisite designs, which provide certain advantages such as aggregating large, heterogeneous samples that increase statistical power and generalisability. However, some limitations of multisite designs are the increased variability of the combined data due to lack of control over conditions, different study designs, high comorbidity, and confounding variables. Perhaps for these reasons, it is difficult to isolate associations between attention biases and specific anxiety disorders such as GAD.

Other studies employed a modified emotional Stroop task to assess attention biases in youth with worry or GAD. One study examined the emotional Stroop effect in children (6–10 years) with high worry, by presenting happy and angry faces in random colours and measuring reaction times and errors to verbal colour-naming responses (Eschenbeck et al. 2004). The results demonstrated that high worriers produced more errors in colour-naming responses, especially in relation to negative emotional faces, in comparison with low worriers. Furthermore, gender did not moderate these effects. Similarly, another study demonstrated a Stroop effect in a clinical sample of children and adolescents with GAD (mean age of 13.47 years old) using positive, neutral, depressed, threat, and trauma-related words (Taghavi et al. 2003). Children and adolescents with GAD showed a strong colour-naming interference effect for negative emotional words relative to the control group, suggesting evidence of a negative attention bias in pathological worriers.

In contrast, Dalgleish et al. (2003) using the same modified Stroop task with emotionally valanced words in a sample of 7—18-year-olds found no significant differences between depressed, GAD, PTSD, or control groups. Furthermore, in the same study, as mentioned above, youth with GAD showed an attention bias for threat on the Dot-probe task, but not on the modified Stroop task. Interestingly, there was no correlation between these two tasks, indicating that they may be measuring different aspects of attention bias. Overall, the results suggest that youth with GAD show an enhanced attention bias towards threat and this effect seems to hold across child and adolescent age groups. However, evidence of a threat bias in youth with GAD appears to be inconsistent in studies that have used the visual Dot-probe task, in particular the studies that have included post-pubertal youth.

#### Interpretation Bias

Six studies assessed the relationship between interpretation bias and worry or GAD in youth (see Table 1). One study (Taghavi et al. 2000) showed that youth with GAD (aged 8–17-year-olds) were more likely to interpret the meaning of ambiguous homograph words (e.g. hang) as threatening as opposed to neutral, in a sentence generation task, whereas non-anxious controls did not show this bias. In younger age groups, two studies examined the content specificity of interpretation biases in children with symptoms of GAD. Bogels, Snieder, and Kindt (2003) presented children (7–12 years) with varying levels of social phobia, separation anxiety, and GAD with ambiguous vignettes describing scenarios related to separation, social, and generalised anxiety. Overall, high anxious children reported the ambiguous scenarios as more dangerous and threatening compared to low anxious controls; however, there was no evidence that children with GAD symptoms showed content specificity for interpretations related to generalised anxiety. The authors suggest that this may be a reflection of comorbidity within the sample and the nature of the disorder, which is characterised by general worry rather than worry concerning specific situations. Similarly, Klein et al. (2018) investigated whether children (7–13 years) with symptoms of GAD showed content-specific interpretation biases for GAD-related words, relative to fear or positive-related words, using a novel auditory interpretation task. In the task, ambiguous auditory stimuli were created by blending emotional words with neutral words that only differed by one phoneme (e.g. thread or threat). Children were allocated to either an open-ended condition, where they had to write down the word that they heard, or a forced choice condition, where they had to select the word out of four possible responses. The study found that children with high levels of GAD selected GAD-related words more often than children with low levels of GAD in the multiple choice condition. However, this finding was not significant in the open-ended condition, suggesting that content specificity is dependent on the research method used to assess interpretation biases. Together, these studies indicate that anxious youth with GAD display a greater tendency to interpret ambiguous information as threatening compared to non-anxious controls, but there are mixed findings for content-specific interpretation biases in children with varying levels of GAD.

These results are consistent with evidence in non-clinical samples of children with varying levels of worry. Suarez and Bell-Dolan (2001) found that 10–12-year-old children with high worry compared to those with low worry, as measured with the Penn State Worry Questionnaire for Children (PSWQ-C), interpreted both ambiguous and threatening scenarios as threatening, expressed more worry in response to the scenario, and exaggerated the probability of future negative events happening to them. In addition, Suarez-Morales and Bell (2006) examined the link between worry, interpretation biases, socio-economic status, and gender in an ethnically diverse community sample of 10–11-year-olds. The study found that worry was associated with negative interpretations of ambiguous and threatening scenarios, whilst stressful life experiences, gender, and socio-economic status played a role in how children processed information. For instance, girls compared to boys tended to generate more adaptive responses and better problem-solving solutions to ambiguous hypothetical scenarios. Finally, a more recent study investigated the various pathways of worry transmission from mothers to their offspring (9–17-year-olds) via interpretation biases (Pasarelu et al. 2017). The study demonstrated that worry in youth was directly associated with interpretation biases towards ambiguous scenarios and maternal worry. Moreover, mother’s interpretation biases influenced interpretation biases in their offspring both directly and indirectly through mother’s expectations of their offspring’s interpretations. Overall, there is clear evidence that indicates interpretation biases are cognitive processes associated with child and adolescent worry in clinical and non-clinical populations.

#### Memory Bias

Only one study investigated the association between memory bias and GAD in youth (see Table 1) and found no differences between youth (7–18-year-olds) diagnosed with GAD, PTSD, depression, and controls, when comparing memory recall of words that were related to threat, depression, trauma, happy, and neutral (Dalgleish et al., 2003). Whilst this study suggests that memory bias was not related to GAD in youth, further research is needed to draw firmer conclusions.

**Table 1:** Summary of studies on information-processing biases associated with child and adolescent worry

Study	*n*	% girls	Age	Sample	Factor	Task	Stimulus used	Outcome measures
Abend et al. (2017)	1291	56	6–18	Mixed clinical and community sample	Attention bias	Dot-probe task	Visual stimuli of angry and neutral faces	SCARED
Bogels et al. (2003)	96	68	7–12	GAD (*n* = 20), SA (*n* = 15), SAD (*n* = 20), Control (*n* = 41)	Interpretation bias	Ambiguous stories	Ambiguous vignettes related to social, separation, and general anxiety scenarios	Ratings and questions related to the interpretation of the scenario
Dalgleish et al. (2003)	93	53	7–18	GAD (*n* = 24), MDD (*n* = 19), PTSD (*n* = 24), Control (*n* = 26)	Attention bias	Dot-probe task	Words related to threat and depression	BPVS; DSRS; RCMAS; The Subject Probability Questionnaire; WORD
						Modified Stroop task	Words related to threat, depression, trauma, positive, and neutral	
					Memory bias	Memory task	Words related to threat, depression, trauma, positive, and neutral	
Eschenbeck et al. (2004)								
Study 1	92	43	7–8	Community	Attention bias	Modified Stroop task	Visual stimuli of angry and happy faces	AFS; WEQ
Study 2	63	59	7–10	Community		Modified Stroop task		AFS; WEQ
Study 3	225	52	6–10	Community		Modified Stroop task		AFS; WEQ
Klein et al. (2018)	666	49	7–13	Community	Interpretation bias	Auditory interpretation task (AIT)	Ambiguous auditory word-blends with GAD-words, negative and positive valenced words	SCARED; Open-ended responses or forced choice responses
Pasarelu et al. (2017)	477	60	9–17	Community	Interpretation bias	Modified version of the Ambiguous Scenarios Questionnaire (ASQ-C; ASQ-M; ASQ-EM)	Ambiguous scenarios with negative or neutral interpretations	PSWQ-C; PSWQ
Roy et al. (2008)	152	47	7–18	Anxious (*n* = 101) [GAD, SP, SAD], Control (*n* = 51)	Attention bias	Dot-probe task	Visual stimuli of angry, happy, and neutral faces	ADIS-IV-C; K-SADS-P; MASC; PARS; SCARED; WISC-III
Suarez and Bell-Dolan (2001)	277	56	10–12	Community	Interpretation bias	The children's opinions of everyday life events (COELE)	Ambiguous and threatening vignettes related to family, relationships, school, performance, health, and personal harm	Ratings and questions related to the interpretation of the scenario
Suarez-Morales and Bell (2006)	292	51	10–11	Community	Interpretation bias	The children's opinions of everyday life events—revised (COELE-R)	Ambiguous and threatening vignettes related to family, relationships, school, performance, health, and personal harm	RCMAS; PSWQ-C; LEC; DHQ
Taghavi et al. (1999)	67	51	9–18	GAD (*n* = 24), Mixed anxiety depression (*n* = 19), Control (*n* = 24)	Attention bias	Dot-probe task	Words related to physical threat, social threat, and depression	BPVS; DSRS; RCMAS; WORD
Taghavi et al. (2000)	57	42	8–17	GAD (*n* = 17), Control (*n* = 40)	Interpretation bias	Homograph sentence generation task	Words related to threat and neutral	BPVS; DSRS; RCMAS; WORD
Taghavi et al. (2003)	38	53	*M* = 14	GAD (*n* = 19), Control (*n* = 19)	Attention bias	Modified Stroop task	Words related to threat, depression, trauma, happy, and neutral	BPVS; DSRS; RCMAS; WORD
Waters et al. (2008)	48	44	7–12	GAD (*n* = 23), Control (*n* = 25)	Attention bias	Dot-probe task	Visual stimuli of happy, angry, and neutral faces	ADIS-C/ADIS-P; SCAS
Waters et al. (2014)	435	52	5–14	GAD (*n* = 75), SAD (*n* = 65), SA (*n* = 18), SP (*n* = 75), Control (*n* = 202)	Attention bias	Dot-probe task	Visual stimuli of happy, angry, and neutral faces	ACQ-C; ADIS-IV-C/P; CASI; SCAS; STAI-C; WISC-R; WRMT-R

*GAD* generalised anxiety disorder, *SAD* social anxiety disorder, *SA* separation anxiety disorder, SP specific phobia, *PTSD* post-traumatic stress disorder, *MDD* major depressive disorder, *ACQ-C* The Anxiety Control Questionnaire-Child, *ADIS-C/ADIS-P* The Anxiety Disorders Interview Schedule for DSM-IV, *AFS* Anxiety Questionnaire for Pupils-Manifests Anxiety subscale, *ASQ* Ambiguous Scenarios Questionnaire (Child/Mother/Mother's expectancy versions), *BPVS* The British Picture Vocabulary Scale, *CASI* Children's Anxiety Sensitivity Inventory, *DSRS* The Depression Self-rating Scale, *K-SADS-P* Schedule for Affective Disorders and Schizophrenia for School-age Children-Present and Lifetime Version, *LEC* The Life Events Checklist, *MASC* Multidimensional Anxiety Scale for Children, *PARS* Paediatric Anxiety Rating Scale for Children, *PSWQ* Penn State Worry Questionnaire (Child/Adult versions), *RCMAS* Revised Children's Manifest Anxiety Scale, *SCARED* Screen for Anxiety and Related Disorders, *SCAS* Spence Children's Anxiety Scale, *STAI-C* State-Trait Anxiety Inventory for Children, *WEQ* Worry Emotionality Questionnaire-Worry and Emotionality subscale, *WISC-III* Wechsler Intelligence Scale for Children-Vocabulary and Block design subscales, *WISC-R* Wechsler Intelligence Scale for Children-Revised, *WORD* The Basic Reading subtest of the Wechsler Objective Reading Dimensions, *WRMT-R* Woodcock Reading Mastery Test-Revised

### Impairment of Executive Functions

#### Attentional Control

Six studies examined the relationship between attentional control and worry or GAD in youth (see Table 2). A common measure of attentional control across studies was the Shift subscale of the Behaviour Rating Inventory of Executive Function—Parent Form (BRIEF). The BRIEF, composed of eight subscales, captures different aspects of executive functions such as inhibition, shifting, emotion control, initiating, working memory, planning, organisation, and monitoring. One study showed that deficits on all eight dimensions of the BRIEF were associated with high worry in 7–12-year-old children (Geronimi et al. 2016). Furthermore, age moderated this relationship for the executive domains of planning, working memory, and monitoring, whilst shifting, inhibition, and emotion control had a more stable association with worry across ages. For planning, working memory, and monitoring, the relationship between worry and executive functioning was much stronger for 7-year-olds than 10-year-olds. By the age of 10, there was minimal difference between high and low worriers in planning, working memory, and monitoring. For other executive functions, including shifting, the higher the reported worry, the poorer the executive functioning across the age range within the sample (7–12-year-olds). Although some executive functions such as working memory, planning, and monitoring were only associated with worry in younger children in the sample (i.e. 7–8-year-olds), a young person’s ability to shift between tasks as reported by their parents was associated with worry for children aged between 7 and 12, suggesting that attentional control is an important cognitive factor for pre-adolescent children.

In line with these findings, one study showed that poor attentional control (shifting) along with emotional control was associated with elevated GAD symptoms in children (7–11 years old) exposed to community violence (Burgers and Drabick 2016). Moreover, these executive functions moderated the relationship between GAD and community violence exposure, where children with poor attentional control and emotional control deficits showed more elevated symptoms of GAD when they were exposed to high levels of direct victimisation. This suggests that improving cognitive control may be useful in reducing pathological worry in vulnerable populations.

Other studies have investigated the importance of attentional control in the cognitive pathways between child temperament and worry or anxiety. One study demonstrated that emotionally reactive child temperaments, described as difficulties in recovering from distress, arousal or excitement, were significantly related to high worry in children (7–10-year-olds) and this relationship was mediated by attentional control and emotional control (Gramszlo and Woodruff-Borden 2015). Extending these findings, another study showed that attentional control and worry serially mediated the relationship between fearful temperaments in children (7–12 years old) and anxiety, whilst worry mediated the association between attentional control and anxiety (Gramszlo et al. 2017). These findings suggest that attentional control and emotion control may be underlying mechanisms linked to worry and the expression of fearful and reactive child temperaments. Furthermore, studies in children and adolescents (mean age 13.6 years old) have shown that GAD symptoms were positively associated with behavioural inhibition and negatively related to attentional control (Sportel et al. 2011). Moreover, attentional control was found to moderate the relationship between behavioural inhibition and symptoms of GAD, which suggests that deficits in attentional control play a role in pathological worry. Similarly, Verstraeten et al. (2011) found that worry was negatively associated with attentional control and inhibitory control and positively correlated with negative affect in 9–13-year-olds. Together, these studies indicate that impairments in attentional control may be an important mechanism underlying the relationship between pathological worry and temperaments such as emotional reactivity, fearfulness, behavioural inhibition, and negative affect. Yet all of the studies on this cognitive factor have employed child samples and limited research has examined the role of attentional control in post-pubertal youths.

#### Working Memory

Four studies examined the relationship between worry and working memory in youth (see Table 2). As mentioned above, one study showed that poor working memory was associated with high worry in children (7–12-year-olds), as measured with the BRIEF questionnaire (Geronimi et al. 2016). However, age moderated this relationship with the effect strongest in younger children (i.e. 7–8-year-olds) and was not present in older ages (i.e. 10–12-year-olds), suggesting that perhaps this cognitive process is underdeveloped in the early stages of childhood and therefore more likely to interfere with worry. Perhaps as children grow older, cognitive capacities in working memory increase with neurocognitive changes in the prefrontal cortex and thus worry may be less likely to interfere with this process as children learn to master certain domains of executive functions.

**Table 2:** Summary of studies on executive functions associated with child and adolescent worry

Study	*n*	% girls	Age	Sample	Factor	Task/questionnaire	Stimulus/subscale	Outcome measures
Burgers and Drabick (2016)	104	50	7–11	Community	Attentional control	BRIEF	Shift	BRIEF; CASI-4R & YI-4; CEQ; FSIQ
					Emotion regulation		Emotion control	
Geronimi et al. (2016)	130	43	7–12	Community	Attentional control	BRIEF	Shift	BRIEF; BAI-Y; BDI-Y; BRIEF; PSWQ-C
					Emotion regulation		Emotion control	
					Working memory		Working memory	
Gramszlo and Woodruff-Borden (2015)	99	40	7–10	Community	Attentional control	BRIEF	Shift	BRIEF; PSWQ-C; TMCQ
					Emotion regulation		Emotion control	
Gramszlo et al. (2017)	152	44	7–12	Community	Attentional control	BRIEF	Shift	BRIEF; BAI-Y; PSWQ-C; TMCQ
Owens et al. (2012)								
Study 1	88	55	12–13	Community				CTAS; NCSAT; RCADS; STAI
Study 2	31	52	12–13	Community	Working memory	Digit span task	Digits	CTAS; NCSAT; RCADS; SATs; STAI; WRAT 4
						Spatial span task	Geometric shapes	
Sportel et al. (2011)	1806	55	*M* = 13.6	Community	Attentional control	ATQ	Attentional control; Effortful control	ATQ; BIS/BAS
Trezise and Reeve (2014)	80	100	14	Community	Working memory	Algebraic working memory task	Alphanumeric symbols and algebraic statements	Algebraic working memory task; Algebraic judgement/worry task; Algebra problem-solving task; FAS; SPM
Trezise and Reeve (2016)	133	30	14	Community	Working memory	Algebraic working memory task	Alphanumeric symbols and algebraic statements	Algebraic working memory task; Algebraic judgement/worry task; Algebra problem-solving task
Verstraeten et al. (2011)	138	53	9–13	Community	Attentional control	ACS		ACS; CDI; CRSQ; ECS; PANAS; PSWQ-C; SCARED-R
						ECS		

*GAD* generalised anxiety disorder, *SAD* social anxiety disorder, *SA* separation anxiety disorder, *ACS* Attentional Control Scale, *ATQ* Adult Temperament Questionnaire, *BAI-Y* Beck Anxiety Inventory-Youth, *BIS/BAS* Behavioural Inhibition System/Behavioural Activation System Scales, *BDI-Y* Beck Depression Inventory—Youth, *BRIEF* The Behaviour Rating Inventory of Executive Function-Parent, *CAPS* The Child and Adolescent Perfectionism Scale, *CASI-4R & YI-4* DSM rating scales for GAD, *CBT* Cognitive Behavioural Therapy, *CDI* Children’s Depression Inventory, *CEMS* Children’s Emotion Management Scales, *CEQ* Community Experiences Questionnaire, *COPE* Cope Inventory, *CRSQ* Children’s Response Styles Questionnaire, *CTAS* Children's Test Anxiety Scale, *DERS* Difficulties in Emotion Regulation Scale, *ECS* Effortful Control Scale, *FAS* The Faces Anxiety Scale, *FSIQ* Full Scale-2 Intelligence Quotient, *NCSAT* National Curriculum Standard Assessment Tests, *PANAS* Positive Affect and Negative Affect Scales, *PSWQ-C* Penn State Worry Questionnaire-Child, *RCADS* Revised Child Anxiety and Depression Scale, *SAS-A* Social Anxiety Scale for Adolescents, *SCARED-R* Screen for Child Anxiety-Related Emotional Disorders-Revised, *SPM* = Raven’s Standard Progressive Matrices, *STAI* The State-Trait Anxiety Inventory, *SATs* school scores based on sub-levels of the Key Stage 2, *TMCQ* = Temperament in Middle Childhood Questionnaire, *WRAT4* The Wide Range Achievement Test–Fourth Edition

In contrast to these findings, two studies with 14-year-olds have found that worry influences working memory capacity in relation to academic performance (Trezise and Reeve 2014, 2016). Working memory was assessed using a novel Algebraic Working Memory task, which involved solving equations and recalling algebraic terms. The studies showed that low working memory was associated with high worry, whilst high working memory was associated with low worry, and this relationship remained stable over time. Furthermore, another study used the digit span and spatial span task and found that high worry interfered with working memory in 12–13-year-olds, which lead to lowered academic performance (Owens et al. 2012). Taken together, the studies reviewed provide evidence that impaired working memory is associated with high worry in children, but few studies have examined these processes in adolescents older than 14 years of age. Furthermore, only one study investigated a child sample and relied on parent report of working memory, which limits the conclusions that can be drawn about the developmental process impacting on the relationship between working memory and worry.

### Verbal Processing of Worry

#### Verbal Worry

Seven studies examined the verbal nature of worry in youth (see Table 3). Two studies used a modified version of The Child and Adolescent Worry questionnaire (CAW) to investigate whether children conceptualise worry as a process related to their fear of negative outcomes or the extent to which they think about negative outcomes (Szabó 2007; Carr and Szabó 2015). Szabó (2007) found that children (mean age of 9 years) reported their worries to be related to both fear and thinking processes, although the extent to which they reported fear of negative outcomes was relatively stronger than the extent to which they think about them, and this was more prominent for physical worries rather than for social worries. Moreover, there was a significant gender difference indicating that females were more likely to worry, fear, or think about social worries, whilst males tended to worry, fear or think about physical worries. In contrast, adults (mean age of 19 years) in this study reported that their worries were more related to thinking processes, suggesting that children and adults may differ in the way they conceptualise their experience of worry.

**Table 3:** Summary of studies on the verbal processing of worry in children and adolescents

Study	*n*	% girls	Age	Sample	Factor	Questionnaire	Subscale	Outcome measures
Carr and Szabó (2015)	93	48	7–12	Community	Verbal worry	CAWS	Fear; Think	CAWS; MCQ-C
Donovan et al. (2017)	114	51	8–12	Community	Cognitive avoidance	WBSI	Thought suppression	CAQ; IUS-C; PSWQ-C; MCQ-C/P; SPSI-RSF
Fialko et al. (2012)	515	53	7–19	Community	Cognitive avoidance	CAQ		^a^CAQ; ^a^IUS; MASC-10; PSWQ-C; ^a^WW2
Frala et al. (2014)	40	52	12–17	Community	Verbal worry	FOVLAS-C	VAS	FOVLAS-C; LRT; PANAS-CN; PSWQ-C; SAM; SUDS
Gosselin et al. (2007)	777	50	12–19	Community	Cognitive avoidance	CAQ	Transformation of images	CAQ; PSWQ-C; WWQ
Laugesen et al. (2003)	528	49	14–18	Community	Cognitive avoidance	WBSI	Thought suppression	PSWQ-C; SPSI-RSF; WAQ; WBSI; WW2
Szabó (2007)	70	51	*M* = 9.13	Community—children	Verbal worry	CAWS	Fear; Think	CAWS; PSOQ
	45	56	*M* = 19.27	Community—adults				

*CAQ* Cognitive Avoidance Questionnaire, *CAWS* The Child and Adolescent Worry Scale-Revised, *LRT* Logical Reasoning Test, *FOVLAS-C* Future-Oriented/Verbal-Linguistic Visual Analog Scale for Children, *IUS* Intolerance of Uncertainty Scale (Child/Parent version), *MASC-10* Multidimensional Anxiety Scale for Children, *MCQ* The Metacognitions Questionnaire (Child/Parent version), *PANAS-CN* Positive and Negative Affect Schedule-Child, *PSOQ* Physical Social Outcome Questionnaire, *PSWQ-C* Penn State Worry Questionnaire-Child version, *SAM* Self-Assessment-Manikin Scales, *SPSI-RSF* Social Problem-Solving Inventory-Revised Short Form, *SUDS* Subjective Units of Distress Scale, *WAQ* Worry and Anxiety Questionnaire, *WBSI* White Bear Suppression Inventory, *WW2* Why Worry II, *WWQ* Why Worry Questionnaire

^a^Brief 5-item measures of the IUS, CAQ and WW2 were created for the study

In line with these findings, Carr and Szabó (2015) showed that children (7–12 years) associated their experience of worry as more closely related to fear of negative outcomes as opposed to thinking about negative outcomes. However, this relationship was moderated by age, as older children tended to report their worries as more strongly associated with thinking and less closely related to fear. Both studies suggest that the self-reported experience of worry, changes throughout different stages of development. Younger children’s experience of worry appears to be more closely associated with fear, but this may evolve to reflect more thinking processes at later stages of cognitive development.

Some studies have found an association between worry and cognitive avoidance (Donovan et al. 2017; Fialko et al. 2012; Gosselin et al. 2007; Laugesen et al. 2003). Cognitive avoidance, typically assessed with self-report questionnaires, is described as an automatic process of avoiding threatening mental imagery and effortful strategies to suppress unwanted thoughts. Two studies employed the Cognitive Avoidance Questionnaire (CAQ) to assess cognitive avoidance strategies used in child and adolescent worry (Fialko et al. 2012; Gosselin et al. 2007). One study of 12–19-year-olds showed that the most common strategies used in adolescent worry were avoidance of stimuli and thought substitution (Gosselin et al. 2007). However, there was no evidence that the cognitive strategy of transforming images into verbal thoughts was associated with worry, suggesting that this strategy is less prevalent and may involve more abstract processes that adolescents are not fully conscious of using. There was no significant gender by age interaction in the various cognitive avoidant strategies used. In another study, cognitive avoidance was found to be moderately associated with worry frequency in child (7–12 years) and adolescent samples (13–19 years) using a brief 5-item measure of the CAQ (Fialko et al. 2012). Furthermore, a gender by age interaction indicated that decreased cognitive avoidance in older age groups was more prominent in boys than girls.

Two studies that have used the White Bear Suppression Inventory (WBSI), a self-report questionnaire measuring the tendency to suppress unwanted thoughts, have produced mixed results in children and adolescents. Consistent with previous findings, Donovan et al. (2017) showed that cognitive avoidance was significantly associated with high worry in 8–12-year-olds. Moreover, parent’s self-report of their own worries, cognitive avoidance strategies, and levels of intolerance of uncertainty were positively associated with child worry. In contrast to these findings, one study in adolescents (14–18-year-olds) showed no association between worry and cognitive avoidance (Laugesen et al. 2003). However, as the WBSI measure has not been validated in adolescent samples, these results should be interpreted with caution. Finally, the only study to utilise an experimental paradigm of a worry induction found no evidence of the verbal-linguistic nature of worry in 12–17-year-old adolescents (Frala et al. 2014). Overall, there was no conclusive evidence for the verbal nature of worry in children and adolescents. However, studies seem to indicate that a child’s conceptualisation and self-reported experience of worry may change throughout different stages of development.

## Discussion

This systematic review examined existing evidence in the youth literature for the three building blocks proposed in Hirsch and Matthews’ cognitive model of pathological worry. Evidence was found that two of the building blocks, negative cognitive biases and deficits in executive functions, are associated with high worry and GAD in children and adolescents. Consistent with the adult literature on cognitive biases, youth with high worry or GAD displayed greater threat interpretations of ambiguous information compared to non-anxious youth, whilst evidence for a threat-related attention bias was mixed and there was limited support for memory bias towards threat in youth. In addition, there was also some evidence that poor attentional control and reduced working memory capacity were associated with high worry or GAD in youth. However, evidence for the verbal processing of worry in children and adolescents was inconclusive. Instead, research indicates that the nature and self-reported experience of worry may change throughout development.

### Summary of Evidence

First, we found partial support that information-processing biases are associated with worry or GAD in children and adolescents. The strongest evidence was for interpretation bias with studies suggesting that the negative or threatening ways children and adolescents interpret ambiguous information are mechanisms associated with pathological worry. On the other hand, evidence for a threat-related attention bias in youth with high worry or GAD was mixed. One reason for this may be the reliability and lack of standardisation of cognitive tasks in attention bias research, such as the use of different paradigms (i.e. Dot-probe task or Stroop task) and task parameters (i.e. pictorial or linguistic stimuli, length of stimulus presentation time, calculation of bias indices, study designs, and comparison groups) that have yielded inconsistent results in child, adolescent, and adult populations (Dudeney et al. 2015; Kruijt et al. 2016; Parsons et al. 2018). For instance, in the current review, one study (Dalgleish et al. 2003) demonstrated that youth with GAD showed an attention bias for threat on the Dot-probe task, but not on a modified Stroop task, with no significant correlations found between the two tasks. The authors suggest that the Dot-probe task may be capturing several aspects of attentional processing, such as vigilance towards the location of a probe at any given time as well as the ability to dwell or shift away from this location (see Fox et al. 2001, for further discussion), whereas the modified Stroop task measures other aspects of attentional processing that show less specificity and may be explained in terms of response competition. Perhaps different cognitive tasks tapping into separate aspects of attentional processing and variations in task parameters may limit the reliability of these measures and result in the mixed findings of attention bias in youth with GAD as it does in adults (Parsons et al. 2018).

Age-related differences in cognitive processing that occur throughout childhood and adolescence may also have led to the mixed findings of attention bias in youth with GAD. A recent meta-analysis on attention biases in anxious youth found that differences in processing threat was moderated by age, with a threat bias more common in younger anxious children (Dudeney et al. 2015). A limitation in paediatric anxiety research is that studies often pool together child and adolescent age groups, making it difficult to disentangle age-related effects. In the current review, no studies were identified that assessed the moderating role of age on attention, interpretation, or memory biases in youth with GAD, highlighting a gap in the literature. Although, it seems that threat-related attention bias as measured by the Dot-probe task is more consistent in studies with younger age groups of pre-adolescent children and early adolescents with GAD (5–13-year-olds), compared to studies where a larger age range was included (7–18-year-olds). In addition, evidence of an attention bias in youth with GAD appeared to be more consistent in Dot-probe studies that used linguistic over pictorial stimuli, suggesting that there may be differences in the processing of threat content in high worriers (i.e. words or imagery). This observation would be in line with a meta-analysis, which found that attention bias in anxious youth was stronger in studies that used linguistic over pictorial stimuli (Dudeney et al. 2015). However, the results are mixed, which indicates that perhaps cognitive developmental factors may influence information-processing biases, with attention bias to threat in youth with GAD more prominent at various stages of development. In summary, the current review provides preliminary support for the importance of the first building block of Hirsch and Matthews’ cognitive model of pathological worry in youth. However, the results are mixed, and we conclude that further investigation of how worry relates to attention, interpretation, and in particular, memory biases is needed.

Second, we found support for the second building block that impaired executive functions were associated with worry in children and adolescents. In line with the adult literature, poor attentional control was related to worry in children and maladaptive child temperaments. One issue we found is that the majority of research has been conducted with pre-adolescent samples and it is unknown whether impairments in attentional control influences worry during adolescence. The review also found that deficits in working memory was a cognitive factor associated with high worry in youth and some evidence suggests that this relationship is moderated by age. Correlational studies demonstrated that poor working memory was more strongly associated with high worry in younger children, but this association was not evident in older children at the age of 10. However, conflicting evidence from experimental studies in adolescents (aged 14) showed that working memory capacity was an important cognitive factor that influenced levels of worry. Broadly, the current review indicates that impairments in executive functions play a role in the process of worry in youth and provides support for the second building block outlined in Hirsch and Matthews’ cognitive model.

Finally, the review found inconclusive evidence for the verbal processing of worry in children and adolescence. We found that age was an important factor associated with worry, as the experience and self-conceptualisation of worry differed throughout various stages of childhood, adolescence, and adulthood. Whilst children’s self-reported experience of worry was related to both fear and thinking processes, the extent to which they fear negative outcomes was relatively stronger than the extent to which they think about them. Adults on the other hand tend to report their worries as more strongly associated with thinking processes. In adolescence, there was limited evidence for the verbal-linguistic nature of worry and the use of cognitive avoidance strategies involving verbal worry. Whilst these studies indicate that a child’s experience of worry involves some level of thinking processes, this may evolve to reflect more salient cognitive processes at later stages of development, and adolescence may be an important transition period where the nature of worry shifts into more thinking like processes, as those typically observed in adult populations.

It is difficult to draw firm conclusions on the exact nature of worry in children and adolescence as no studies have directly investigated whether the worry process in youth involves more verbal or more imagery-based worry. It is also unclear at what age worry acquires the characteristics of verbal thoughts and its associated cognitive avoidance response. Even though Vasey (1993) proposed that the transition from mental imagery to verbal worry develops from middle childhood onwards, no studies have directly investigated this hypothesis.

In addition to developmental changes, one of the challenges in understanding youth worry is the reliability of assessment tools that measure worry in children and adolescents (Cartwright-Hatton et al. 2011). For instance, worry questionnaires in youth are often drawn from adult conceptualisations of worry and anxiety. Thus, items on a self-report questionnaire may reflect more complex worry-related cognitive processes that children may not fully comprehend (Smith and Hudson 2013). This may explain the inconclusive evidence regarding the third component of Hirsch and Matthews’ cognitive model of worry in youth. Whilst some studies suggest that youth may experience worry in verbal form or thinking processes, the nature of worry appears to change across different stages of development, and methodological issues with the measurement of youth worry casts doubt on these findings.

### Future Directions

Hirsch and Matthews’ model of pathological worry (2012) has yet to be directly tested with children and adolescents. It is therefore not clear whether these cognitive factors, which have been well supported in the adult literature, also operate during child and adolescent worry and at what developmental stage they begin to play a role. Testing the three components of this model in a large sample of young people is an important focus for future research. In addition, significant work is required to improve the reliability of the assessment of information-processing biases in youth. This field would benefit from the development of standardised reliable measures so that meaningful comparisons can be drawn across studies, especially in relation to attention bias research where reliability problems have been identified. A further problem is that the majority of research on the cognitive mechanisms associated with child and adolescent worry has been cross-sectional. Future studies employing experimental as well as longitudinal designs would provide a deeper insight into the causal pathways underlying pathological worry in youth and shed light on the cognitive mechanisms to target for early interventions and treatments.

Furthermore, the assessment of child and adolescent worry has relied on retrospective self-reports, questionnaires, vignettes, and interviews to assess worry, often derived from adult conceptualisations of worry. Future research developing validated measures of worry that reflect child relevant concepts are needed for more appropriate assessment of worry in youth that can be easily interpreted and comprehended. A large body of research in adults has utilised worry induction paradigms to examine the consequences of active worry, which have contributed to a greater understanding of the worry processes. Perhaps, the use of worry induction paradigms to investigate the direct impact of active worry on cognitive functions may address some limitations of using retrospective self-report questionnaires in youth.

**Fig. 2:**
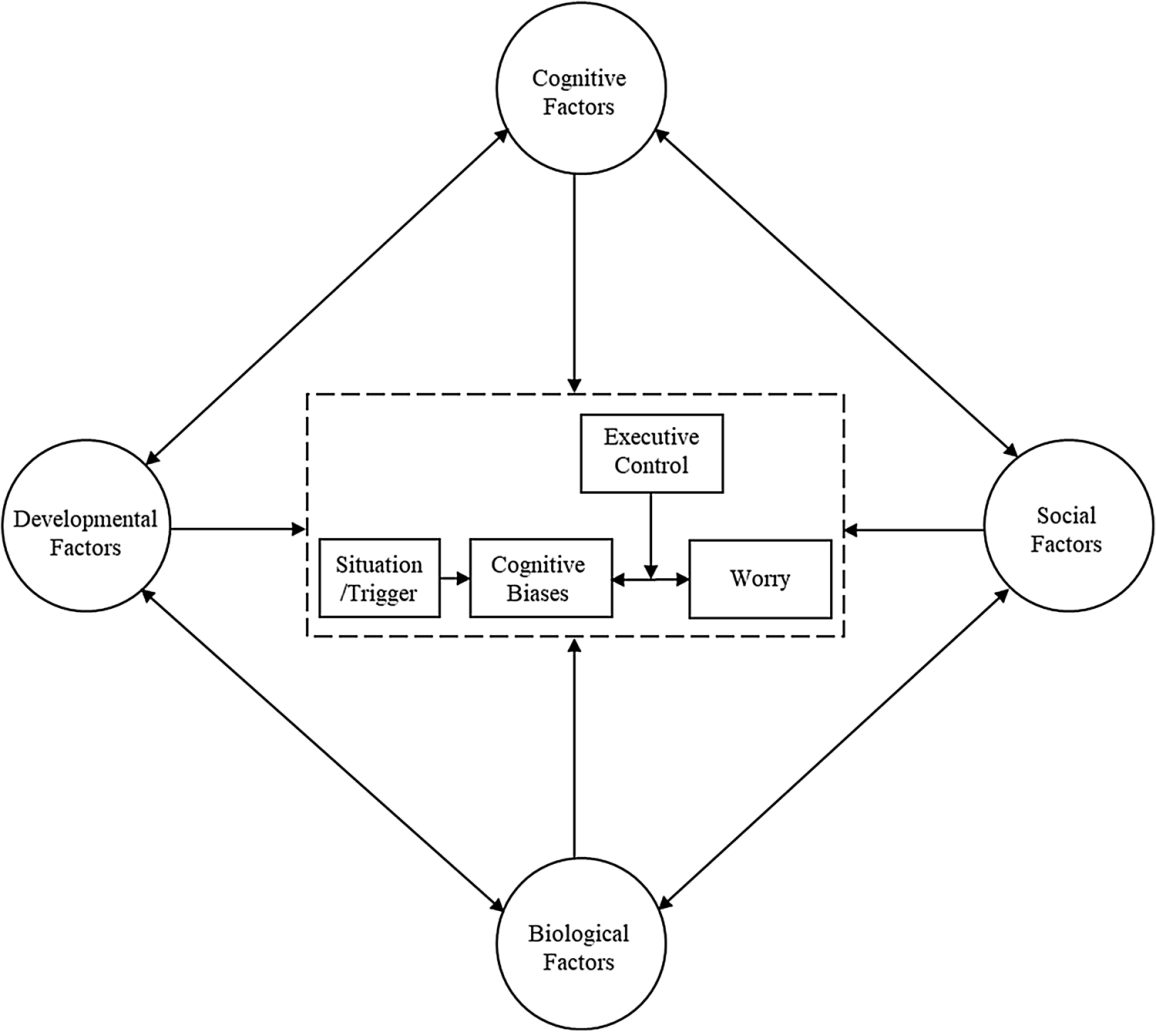
Cognitive model of child and adolescent worry

Finally, an important direction for future research investigating worry in children and adolescence is to investigate the cognitive mechanisms of worry within a developmental framework, taking into consideration developmental changes, which may influence the nature and experience of worry in youth. Based on the mixed findings in this review, we suggest that developmental factors may be an important component that influences the role of information-processing biases, executive functions, and verbal processing of worry in the developmental trajectory of pathological worry in children and adolescents. However, few studies have directly examined the moderating role of age in the development of worry in youth, highlighting an important gap in the literature. Future research should aim to investigate the impact of age or assess other developmental markers such as level of cognitive development attained through conservation and logical reasoning tasks (Frala et al. 2014), or questions regarding puberty (Caes et al. 2016), which may have an important influence on the development of worry in youth. Integrating a developmental framework would emphasise that a child is continuously changing and evolving over time and would provide a better understanding of the trajectory of worry across childhood and adolescence. Therefore, to facilitate these important gaps in the literature and a framework for future studies, we propose a cognitive developmental model of child and adolescent worry described below and illustrated in Fig. 2.

### A Cognitive Model of Child and Adolescent Worry

Based on the findings of our systematic review, we propose an extension of Hirsch and Mathews’ cognitive model (2012) that incorporates cognitive, developmental, biological, and social factors that are likely to impact the development of worry and GAD in youth. Our cognitive model of child and adolescent worry proposes that negative cognitive biases and deficits in executive control processes are central components contributing to worry or GAD in youth (see Fig. 2) as in adult worry (Hirsch and Matthews 2012). However, the evidence suggests that the verbal nature or worry may not be as critical in young people as in adults, although this is something that requires further research. Our proposed model of youth worry allows for testable hypotheses that may help explain the complex interplay between a range of factors associated with worry or GAD in youth.

Central to the cognitive model is the role of cognitive biases and executive control. Building upon previous data and theory, we hypothesise that worry is preceded by a situation or an initial trigger, which may be internal, such as an intrusive thought, or an external event such as an everyday stressor. When a certain situation or trigger arises, involuntary ‘bottom-up’ processes, such as attention biases towards threatening cues, negative interpretation biases of ambiguous information, or negative memory biases, lead to the heightened representation of threat initially entering into awareness. Subsequently, ‘top-down’ executive control processes, such as attentional control or working memory, interact with these cognitive biases to determine whether a worry episode manifests. Developmental, social, biological, and other cognitive factors are also incorporated into the model to provide a framework to explicate the vital components that may influence the development of worry throughout childhood and adolescence.

Negative information-processing biases, which typically occur without awareness and are typically involuntary, are likely to be re-enforced over time through habitual thought patterns and become activated when future triggers are encountered. In our model, a worry-prone child or adolescent may have the tendency to make threatening interpretations and are likely to direct their attention towards potentially negative outcomes when faced with an ambiguous situation. This direct relationship between negative cognitive biases and worry is yet to be examined in youth populations, as the majority of research has focused on anxiety disorders. Future studies should aim to investigate the role of cognitive biases on the transdiagnostic factor of child and adolescent worry. Research in sub-clinical samples is especially important in order to examine the trajectory of worry before it manifests into a pathological state, as seen in GAD.

In the second component of our model, we hypothesise that deficits in executive control impairs children and adolescent’s ability to redirect attention away from negative intrusions. In line with Hirsch and Mathews’ (2012) cognitive model, we propose that high worriers may have insufficient executive control to override negative intrusions and are less able to redirect their thoughts away from negative information once it enters into awareness. We hypothesise that ‘top-down’ executive control processes, such as attentional control or working memory, moderates the association between cognitive biases and worry. For instance, in low worriers, when a representation of threat enters into awareness through ‘bottom-up’ influences of cognitive biases, children and adolescents with greater executive control are able to inhibit the negative intrusion from developing into a worry episode. In contrast, poor executive control in high worriers make it difficult to ignore negative intrusions, which leads to uncontrollable and repetitive worry. Future experimental studies investigating the impact of active worry on executive functions such as working memory capacity or attentional control would help to identify how executive control processes contribute to worry.

Third, the model outlines the importance of developmental, biological, social, and other cognitive factors that may contribute to worry in children and adolescents. These factors provide a framework in which cognitive biases and executive control processes are influenced in complex ways, and aims to capture some of the complexity of the development of worry and GAD in youth. In our cognitive model, we propose that developmental factors (e.g. age, puberty or learning abilities), biological factors (e.g. genetics or temperament), social factors (e.g. interacting with peers, establishing and maintaining relationships, social support, family or parental relationships), and other cognitive factors (e.g. intolerance of uncertainty, metacognitive beliefs, problem-solving capabilities or emotion regulation) all have a large impact on the development of cognitive biases, executive functions, and how worry is experienced by children and adolescents. In our model, cognitive, developmental, biological, and social factors are interrelated and are an important framework for understanding the way cognitive biases and executive control operate and become habitual patterns over time.

Taking a developmental approach is especially important. To the best of our knowledge, no studies have examined how executive functions or attention, interpretation, and memory biases develop throughout childhood and adolescence in relation to outcomes of worry. One correlational study has shown that age moderates the relationship between worry and executive functions in children (Geronimi et al. 2016), whilst recent reviews indicate that the association between negative interpretation biases and anxious youth increases with age (Stuijfzand et al. 2017) and negative attention biases in anxious children varies across childhood (Dudney et al. 2015). Future longitudinal studies investigating how cognitive biases and executive functions evolve over time would provide a deeper understanding of the developmental factors that impact the nature of worry in children and adolescents. Measurement of many of these complex cognitive processes rely on children being able to accurately report their experiences and are often dependent on measures that younger children in particular to do not fully comprehend (Smith & Hudson, 2013). Progress in this field has been further limited by the absence of studies with sufficient power to test for possible differences in these processes across development. Future studies need to include large sample sizes of youth of varying ages to allow the examination of how these processes are related to worry across development.

In line with the findings of the current review, we hypothesise that negative intrusions in youth may take the form of thoughts, images, or impressions depending on developmental and cognitive factors such as the child’s age, cognitions, or level of cognitive abilities attained. Further research on whether worry in youth is primarily a verbal process or imagery based would also provide more targeted approaches to early interventions for worry and improve current treatments. This illustrates that cognitive models of adult worry may not be fully appropriate for younger populations and a developmentally sensitive model of worry in children and adolescents is needed.

In summary, we propose that negative cognitive biases and deficits in executive control facilitate the development of worry in children and adolescents. These cognitive vulnerability factors interact with each other and are likely to change over time, with the influence of cognitive, biological, social, and developmental changes having different degrees of influence across different periods of childhood and adolescence. Our proposed model focuses primarily on cognitive elements of worry and information-processing components building upon a cognitive model of adult pathological worry (Hirsch and Matthews 2012). We fully acknowledge that there are other cognitive theories and many cognitive, biological, parental, and environmental factors that play an important role in child and adolescent worry. In this review, we have focused on cognitive biases and executive functions due to the limited research that has investigated these factors in relation to worry or GAD in youth. Our proposed cognitive model is designed to provide a deeper understanding of the cognitive pathways associated with pathological worry, across child and adolescent development, as well as insights into the protective factors that may contribute to resilience to worry in youth.

### Strengths and Limitations

One limitation of the current review and the literature, in general, is that the majority of studies are cross-sectional and do not address the causal direction between worry and the cognitive processes of interest. Another limitation is that the studies reviewed often combine a wide age range of children and adolescents, making it difficult to disentangle age-related effects, and it may be that some cognitive processes are not fully developed in certain age groups. Therefore, it is challenging to draw specific conclusions as to how worry may change across childhood and adolescence, with few studies examining narrow age ranges in youth.

On the other hand, the review has many strengths that add to the literature. To the best of our knowledge, this is the first systematic review to evaluate the applicability of Hirsch and Matthews’ cognitive model of worry to younger populations. Based on the results of the systematic review, we proposed a new cognitive model of child and adolescent worry intended to provide a theoretical framework to help guide future research on the underlying mechanisms that play a role in worry in youth. These pathways provide testable hypothesis and a research framework for future studies, which may help to identify the mechanisms to target in early interventions and treatments for worry. Whilst our model focuses on the cognitive elements and information-processing mechanisms of worry in youth, the model also acknowledges wider social and environmental maintenance influences that play an important role in maintaining worry. Examining the role of maintenance factors is an important direction for future research and would provide a greater insight into the development and maintenance of worry in youth.

## Conclusion

Hirsch and Matthew’s cognitive model of pathological worry provides an evidence- based framework for investigating information-processing, executive functions, and verbal worry in the development of child and adolescent worry. We found evidence that some elements of Hirsch and Matthews’ cognitive model were applicable to understanding worry in younger populations; however, certain cognitive processes may still be developing in children and adolescence, and the model as a whole may not capture these developmental nuances and complexities. Therefore, as we have proposed in our cognitive model of child and adolescent worry, it is crucial that future research incorporates a developmental approach to understanding the cognitive mechanisms underlying worry, which remains a largely unexplored area of empirical research. In particular, longitudinal studies and experimental designs testing causal hypotheses would provide useful insights into the causal and maintaining factors of worry in youth and how these processes interact and change over time. Childhood and adolescence is a critical developmental period that entails major cognitive, emotional, social, and physiological changes. Understanding this phenomenon in children and adolescents is imperative for building emotionally resilient and healthy individuals well into adulthood.

## Appendix

See Table 4.

**Table 4:** Summary of quality ratings of studies in the systematic review

Studies	Sections						
	A	B	C	D	E	F	Global rating
Abend et al. (2017)	Strong	Moderate	Strong	Moderate	Strong	N/A	Strong
Bogels et al. (2003)	Strong	Moderate	Moderate	Moderate	Strong	N/A	Strong
Burgers and Drabick (2016)	Strong	Moderate	Strong	Moderate	Strong	N/A	Strong
Carr and Szabó (2015)	Strong	Moderate	Moderate	Moderate	Strong	N/A	Strong
Dalgleish et al. (2003)	Strong	Moderate	Strong	Moderate	Strong	N/A	Strong
Donovan et al. (2017)	Strong	Moderate	Moderate	Moderate	Strong	N/A	Strong
Eschenbeck et al. (2004)	Strong	Moderate	Moderate	Moderate	Strong	N/A	Strong
Fialko et al. (2012)	Strong	Moderate	Strong	Moderate	Strong	N/A	Strong
Frala et al. (2014)	Strong	Strong	Strong	Strong	Strong	N/A	Strong
Geronimi et al. (2016)	Strong	Moderate	Strong	Moderate	Strong	N/A	Strong
Gosselin et al. (2007)	Strong	Moderate	Strong	Moderate	Strong	N/A	Strong
Gramszlo and Woodruff-Borden (2015)	Strong	Moderate	Weak	Moderate	Strong	N/A	Moderate
Gramszlo et al. (2017)	Strong	Moderate	Weak	Moderate	Strong	N/A	Moderate
Klein et al. (2017)	Strong	Strong	Moderate	Moderate	Strong	N/A	Strong
Laugesen et al. (2003)	Strong	Moderate	Strong	Moderate	Strong	N/A	Strong
Owens et al. (2012)	Strong	Moderate	Weak	Moderate	Strong	N/A	Moderate
Pasarelu et al. (2017)	Strong	Moderate	Moderate	Moderate	Strong	N/A	Strong
Roy et al. (2008)	Strong	Strong	Strong	Strong	Strong	N/A	Strong
Sportel et al. (2011)	Strong	Moderate	Moderate	Moderate	Strong	N/A	Strong
Suarez and Bell-Dolan (2001)	Strong	Moderate	Strong	Moderate	Strong	N/A	Strong
Suarez-Morales and Bell (2006)	Strong	Moderate	Strong	Moderate	Strong	N/A	Strong
Szabó (2007)	Strong	Moderate	Moderate	Strong	Strong	N/A	Strong
Taghavi et al. (1999)	Strong	Strong	Strong	Moderate	Strong	N/A	Strong
Taghavi et al. (2000)	Strong	Strong	Strong	Moderate	Strong	N/A	Strong
Taghavi et al. (2003)	Strong	Strong	Strong	Moderate	Strong	N/A	Strong
Trezise and Reeve (2014)	Strong	Moderate	Strong	Moderate	Strong	N/A	Strong
Trezise and Reeve (2016)	Strong	Moderate	Strong	Moderate	Strong	N/A	Strong
Verstraeten et al. (2011)	Strong	Moderate	Moderate	Moderate	Strong	N/A	Strong
Waters et al. (2008)	Strong	Strong	Strong	Moderate	Strong	N/A	Strong
Waters et al. (2014)	Strong	Strong	Strong	Moderate	Strong	N/A	Strong

Quality assessment tool (Effective Public Health Practice Project 1998)

*A* selection bias, *B* study design, *C* confounders, *D* blinding, *E* data collection methods, *F* withdrawals/drop-outs at follow-up, *3-point rating scale* strong/moderate/weak
